# Right kidney contusion following endoscopic mucosal resection of hepatic flexure lymphangioma: a rare case report

**DOI:** 10.3389/fsurg.2026.1731412

**Published:** 2026-05-28

**Authors:** Lexin Liu, Zehui Wei, Li Liu, Haizhou Wang, Fengjun Jiang, Guoxin Huang

**Affiliations:** 1 Department of Gastroenterology and Endoscopy, Shenzhen Hospital (Fu Tian) of Guangzhou University of Chinese Medicine, Shenzhen, Guangdong, China; 2 Department of Gastroenterology and Endoscopy, Guangdong Provincial Hospital of Chinese Medicine, Guangzhou, Guangdong, China

**Keywords:** conservative management, endoscopic mucosal resection, hepatic flexure lymphangioma, postoperative complications, renal injury, right kidney contusion

## Abstract

Endoscopic mucosal resection (EMR) is widely utilized for the removal of benign colonic lesions; nevertheless, it carries certain inherent risks. We present a rare case of right kidney contusion following EMR for hepatic flexure lymphangioma, representing an uncommon and underreported complication. A 50-year-old woman underwent colonoscopy, which identified a 3 cm cystic mass at the hepatic flexure, subsequently diagnosed as a lymphangioma. EMR was performed with electrocoagulation and mechanical clamping. Postoperatively, she developed progressively worsening upper abdominal pain but did not present with hematuria or hematochezia. Imaging demonstrated edema of the ascending colon wall, free pericolic gas, a perirenal hematoma, and a hemorrhagic focus in the liver. She was managed conservatively with antibiotics, supportive care, and close monitoring in the intensive care unit. Over the following two weeks, her symptoms improved substantially and she was discharged without sequelae. This case illustrates that rare complications such as renal contusion may occur after EMR, emphasizing the need for vigilant postoperative care and awareness of potential injury to adjacent organs, particularly the kidneys. Timely imaging and a multidisciplinary approach are essential for optimal outcomes.

## 1 Introduction

Endoscopic mucosal resection (EMR) is frequently employed for the removal of benign colonic lesions, with advantages such as minimal invasiveness, shorter recovery time, and reduced hospital stays compared with conventional open surgery (1, 2). This technique has been widely adopted and generally achieves favorable outcomes; however, it is not entirely without risk. Although complications are relatively uncommon, when they occur they may present with diverse clinical phenotypes and in some cases lead to significant morbidity.

Right kidney contusion represents an exceptionally rare complication following EMR of colonic lesions, particularly when the lesion is located at the hepatic flexure, which lies in close anatomical proximity to the right kidney (3). Hepatic flexure lymphangiomas themselves are rare benign tumors that can be challenging to diagnose due to their nonspecific clinical manifestations (4). The anatomical relationship of the hepatic flexure with adjacent retroperitoneal structures, especially the kidneys, places this region at greater risk for inadvertent injury during endoscopic resection (5).

In this report, we describe a case of right kidney contusion occurring after EMR of a hepatic flexure lymphangioma. The unusual nature of this complication provides an opportunity to remind clinicians that although EMR is considered safe, unexpected extraintestinal injuries may arise in anatomically complex regions. Patients with renal injury following EMR may present only with vague or nonspecific abdominal pain, with clinical findings limited to localized tenderness. In addition, classical manifestations of renal injury such as hematuria, flank pain, or hemodynamic instability (6) may be absent, especially in cases of minor renal contusion or retroperitoneal hematoma. In such cases, cross-sectional imaging plays a critical role, as it may reveal hematoma, perirenal edema, or other subtle signs of retroperitoneal involvement that are not evident on physical examination.

## 2 Case description

A 50-year-old female with no past medical history presented with a six-day history of constant upper abdominal pain. The pain was described as a dull ache located in the upper right abdomen, without radiation, nausea, vomiting, or change in bowel habits. She denied hematuria, hematochezia, or other systemic symptoms. On examination, the patient was afebrile, with localized tenderness in the upper right abdomen but no signs of peritonitis. Liver and renal function tests were within normal limits, and abdominal ultrasound revealed no acute pathology. Colonoscopy was subsequently performed and revealed a 3 cm cystic mass occupying the hepatic flexure of the colon. Given its cystic appearance and imaging features, the lesion was suspected to be a hepatic flexure lymphangioma. Histopathological examination of biopsy specimens confirmed the diagnosis of lymphangioma, a rare benign tumor of lymphatic origin. Following multidisciplinary discussion, excision of the lesion by EMR was planned, and informed consent was obtained.

**Figure 1:**
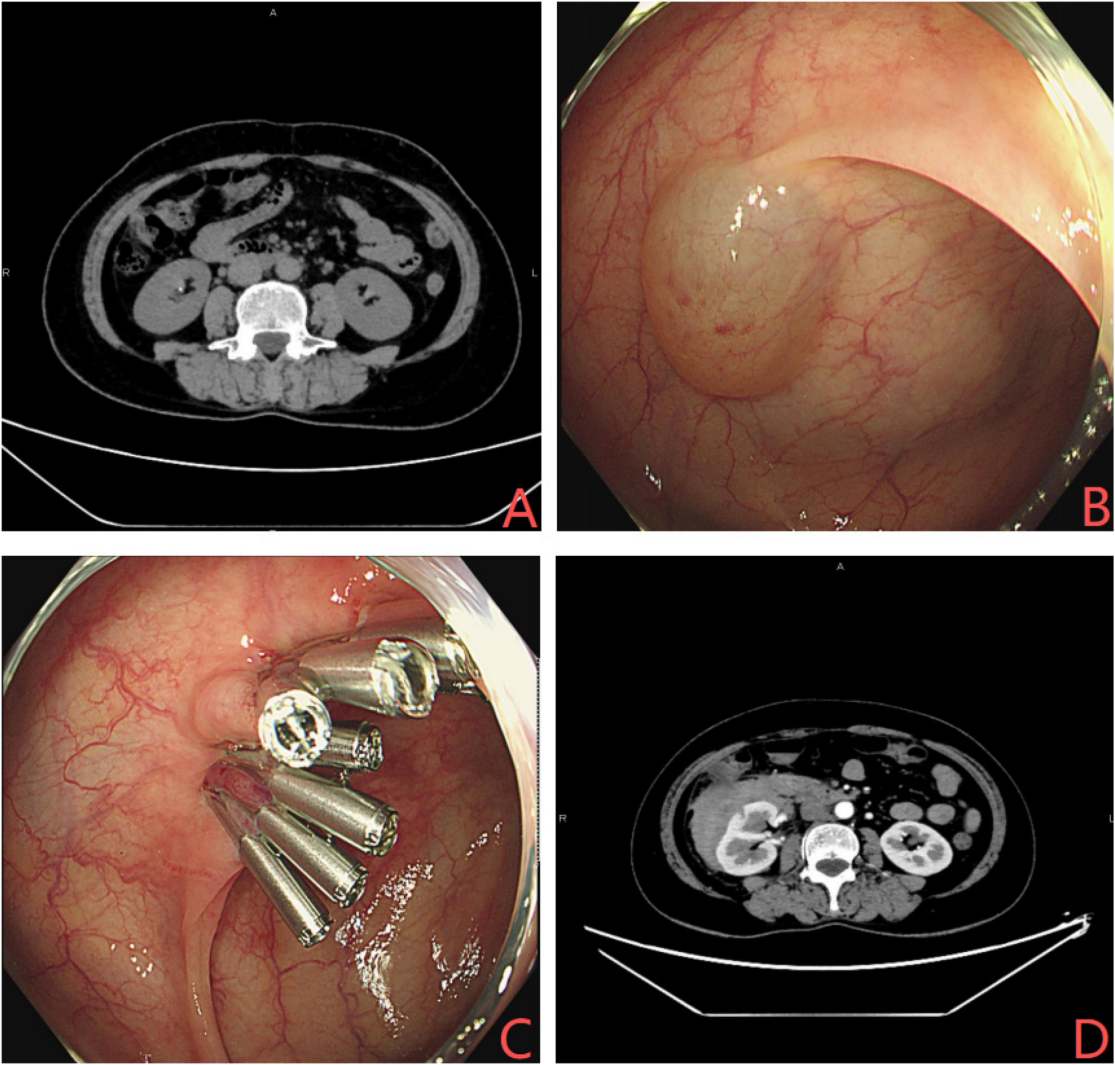
Imaging and procedural findings in a 50-year-old woman with right kidney contusion following EMR for hepatic flexure lymphangioma. **(A)** CT images of the chest and upper abdomen show liver cysts and small kidney stones in the right and left kidney. **(B)** Colonoscopy image shows 3 cm cystic mass within the hepatic flexure suggestive of lymphangioma. **(C)** Intraoperative image showing repeated electrocoagulation and titanium clamp used for resection of lesion. **(D)** Postoperative contrast-enhanced CT image shows ascending colon wall edema, pericolic free gas, perirenal hematoma, and hemorrhagic focus in the right kidney cortex suggestive of right kidney contusion.

The procedure was performed under general anesthesia using electrosurgical techniques. The lesion was first treated with electrocoagulation to control potential bleeding and then excised using mechanical clamping. The resection was completed without immediate complications, and the patient was closely monitored postoperatively. Initially, she remained stable, but 24 h later she developed progressively worsening upper abdominal pain, although she continued to deny hematuria, hematochezia, or other signs of infection. Due to worsening symptoms, a contrast-enhanced abdominal CT scan was obtained to evaluate possible complications. The imaging demonstrated marked edema of the ascending colon wall, free pericolic gas, and a perirenal hematoma involving the right kidney. Additionally, a hemorrhagic focus within the anterolateral cortex of the right kidney was consistent with renal contusion (Figure 1), representing a rare complication following EMR.

Given the severity of findings, the patient was transferred to the intensive care unit (ICU) for close observation and conservative management. She was treated with intravenous antibiotics, analgesia, hydration, and nutritional support, while serial imaging was performed to monitor the progression of the hematoma. During ICU stay, the patient remained hemodynamically stable, with no episodes of hypotension or tachycardia requiring intervention. Serial laboratory monitoring demonstrated that hemoglobin levels fluctuated between 106 and 119 g/L without significant decline, suggesting the absence of ongoing active bleeding. White blood cell counts ranged from 7.01 to 11.26  ×  10⁹/L, while C-reactive protein(CRP) levels initially increased (37.92–104.54 mg/L) and subsequently decreased with treatment. Renal function remained within normal limits throughout hospitalization. Over the next two weeks, her abdominal pain improved, the hematoma gradually resolved, and no further complications occurred. Following transfer to the general ward, hemoglobin levels progressively increased to 134 g/L, and inflammatory markers normalized (CRP decreased to 3.23 mg/L), indicating resolution of inflammation and stabilization of the hematoma. Follow-up imaging confirmed resorption of the perirenal hematoma, and the patient was discharged in good condition without sequelae.

## 3 Discussion

EMR has emerged as the standard technique for resecting superficial gastrointestinal lesions due to its minimally invasive nature and lower associated morbidity compared with surgical resection (7). Its efficacy in the removal of superficial colorectal cancer, polyps, and other benign lesions has been well established (8). Although complications are uncommon, they may be clinically significant, particularly when procedures are performed in anatomically high-risk regions.

Although renal contusion following EMR has not been widely reported, several studies have described extraintestinal complications associated with endoscopic resection, particularly in anatomically complex regions. Reported adverse events include retroperitoneal perforation, post-polypectomy electrocoagulation syndrome, and thermal injury to adjacent organs such as the small intestine and mesentery (9–11). These complications share a common mechanism involving deep mural injury or excessive thermal spread beyond the intestinal wall. Compared with these reports, our case is unique in demonstrating renal involvement, likely due to the close anatomical relationship between the hepatic flexure and the right kidney. This further expands the spectrum of potential extraintestinal injuries associated with EMR and highlights the need for heightened awareness when operating in retroperitoneal-adjacent segments of the colon.

We report an unusual adverse event of right kidney contusion following EMR of a hepatic flexure lymphangioma, broadening the spectrum of injuries that may occur after colon lesion resection and underscoring the potential anatomical risks associated with this procedure. The hepatic flexure is closely related to the right kidney and the duodenum and surrounded laterally by retroperitoneal structures, making it a particularly vulnerable site for EMR (5). The close proximity of these organs increases the risk of inadvertent injury. 'As previously reported by Roy et al., thermal spread from electrocoagulation can extend beyond targeted tissues, potentially damaging adjacent organs (12). In this case, repeated electrocoagulation for hemostasis, combined with the lesion's size and location, likely contributed to thermal injury that extended through the colonic wall into retroperitoneal tissues, leading to perirenal hematoma (13) and right renal contusion (Figure 1D). Although rare, this mechanism is consistent with other reports (11, 14) of unintended thermal injury during endoscopic procedures. Given the anatomical configuration of the right mesocolon, where the colon attaches to the retroperitoneum, heat generated during resection could readily affect adjacent structures such as the kidney, liver, and duodenum.

Colonic lymphangiomas, although rare, have been reported in multiple regions of the colon, most frequently in the cecum and ascending colon, while hepatic flexure lesions remain particularly uncommon. As Lee et al. noted, these tumors are usually asymptomatic but may present with vague abdominal complaints or even intussusception when larger (15). EMR has generally been considered safe and effective for resecting such tumors, with low recurrence rates and favorable outcomes (16). Nevertheless, our case emphasizes the complexity of performing EMR in anatomically challenging locations, where risks of collateral injury are inherently higher. Excessive electrocoagulation during resection may increase the likelihood of unintended thermal spread, a complication also highlighted by Ochi et al. in their evaluation of post-EMR electrocoagulation syndrome (17).

Renal contusions or other retroperitoneal injuries may be difficult to detect postoperatively, as early clinical manifestations are often nonspecific. As Chang et al. described, perirenal hematomas may present with subtle or ambiguous signs and can remain undiagnosed without cross-sectional imaging (18). In our patient, there were no clinical signs of perforation or infection, and the renal injury was only discovered after CT was performed due to worsening abdominal pain. The presence of pericolic free gas also suggested possible microperforation, potentially providing a pathway for gas to spread into retroperitoneal spaces. In this situation, timely imaging was crucial for detection and management.

Iatrogenic renal contusions are rare but, when recognized early, can often be managed conservatively. Stable patients without active hemorrhage or hemodynamic compromise are generally treated successfully with supportive care, including analgesia, antibiotics, hydration, and serial imaging (19). This aligns with recent literature emphasizing the effectiveness of conservative management for minor renal injuries in the absence of complicating factors.

This case carries several important clinical implications. First, postoperative follow-up is essential, particularly for patients who develop unexplained symptoms, as rare complications may initially mimic benign postoperative discomfort. Second, preoperative imaging with high-resolution CT or endoscopic ultrasound can help delineate lesion depth and proximity to critical structures, thereby guiding the safest therapeutic approach. Third, preventive strategies should focus on minimizing thermal injury during resection. In addition to limiting the depth and duration of electrocoagulation, newer endoscopic techniques may offer safer alternatives. Underwater EMR (UEMR), for example, allows resection to be performed in a water-filled lumen without submucosal injection, which both reduces the need for coagulation and provides a heat-sink effect that limits thermal spread. UEMR has been increasingly recognized as a safe and effective alternative for colorectal lesion resection, particularly for lesions in difficult locations (20, 21). Similarly, cold resection techniques, including cold snare polypectomy and cold EMR, avoid electrocautery altogether, thereby eliminating the risk of thermal damage. These techniques have demonstrated excellent safety profiles with minimal risk of perforation and delayed bleeding (22). Their use may be considered for selected benign lesions in anatomically high-risk locations to minimize collateral injury.

Looking ahead, integrating these techniques into routine practice, particularly in anatomically challenging locations, will require further comparative studies to evaluate safety, efficacy, and long-term outcomes. Future research should also explore hybrid approaches that combine cold resection with selective limited coagulation for hemostasis, as well as the role of novel energy devices designed to minimize lateral heat spread. By adopting such innovations, endoscopists may significantly lower the risk of extraintestinal complications while maintaining the effectiveness of minimally invasive resection.

In conclusion, although EMR remains a highly effective and minimally invasive treatment for benign gastrointestinal lesions, clinicians must remain aware of the full spectrum of potential complications, including rare extraintestinal injuries. When operating near the kidney, a comprehensive approach that integrates careful preoperative planning, judicious selection of resection techniques such as UEMR or cold resection, and vigilant postoperative monitoring is paramount to minimizing risk and optimizing patient outcomes.

## Data Availability

The original contributions presented in the study are included in the article/Supplementary Material, further inquiries can be directed to the corresponding author.
